# *Streptococcus mutans*-derived extracellular matrix in cariogenic oral biofilms

**DOI:** 10.3389/fcimb.2015.00010

**Published:** 2015-02-13

**Authors:** Marlise I. Klein, Geelsu Hwang, Paulo H. S. Santos, Osvaldo H. Campanella, Hyun Koo

**Affiliations:** ^1^Center for Oral Biology, University of RochesterRochester, NY, USA; ^2^Biofilm Research Lab, Levy Center for Oral Health, Department of Orthodontics and Divisions of Pediatric Dentistry and Community Oral Health, School of Dental Medicine, University of PennsylvaniaPhiladelphia, PA, USA; ^3^Whistler Center for Carbohydrate Research, Purdue UniversityWest Lafayette, IN, USA

**Keywords:** biofilms, dental caries, *Streptococcus mutans*, extracellular matrix, spatial heterogeneities, mechanical stability, exopolysaccharides, eDNA

## Abstract

Biofilms are highly structured microbial communities that are enmeshed in a self-produced extracellular matrix. Within the complex oral microbiome, *Streptococcus mutans* is a major producer of extracellular polymeric substances including exopolysaccharides (EPS), eDNA, and lipoteichoic acid (LTA). EPS produced by *S. mutans*-derived exoenzymes promote local accumulation of microbes on the teeth, while forming a spatially heterogeneous and diffusion-limiting matrix that protects embedded bacteria. The EPS-rich matrix provides mechanical stability/cohesiveness and facilitates the creation of highly acidic microenvironments, which are critical for the pathogenesis of dental caries. In parallel, *S. mutans* also releases eDNA and LTA, which can contribute with matrix development. eDNA enhances EPS (glucan) synthesis locally, increasing the adhesion of *S. mutans* to saliva-coated apatitic surfaces and the assembly of highly cohesive biofilms. eDNA and other extracellular substances, acting in concert with EPS, may impact the functional properties of the matrix and the virulence of cariogenic biofilms. Enhanced understanding about the assembly principles of the matrix may lead to efficacious approaches to control biofilm-related diseases.

Biofilms are highly dynamic and structured communities of microbial cells that are firmly attached to a surface and enmeshed in a three-dimensional (3D) extracellular matrix of polymeric substances such as exopolysaccharides (EPS), proteins and nucleic acids (Branda et al., 2005; Flemming and Wingender, 2010). The extracellular matrix produced by specific microorganisms promotes microbial adhesion and cohesion while also hindering diffusion (Flemming and Wingender, 2010). It essentially provides a 3D scaffold for biofilm development, helping to shape spatial, metabolic and microenvironmental heterogeneities (Stewart and Franklin, 2008; Mann and Wozniak, 2012; Wozniak and Parsek, 2014). Thus, the matrix is critical for the existence of biofilm lifestyle and full expression of virulence by bacterial and fungal pathogens. Consequently, matrix builders (i.e., the microorganisms that produce or process polymeric substances) play a key role in the development of biofilms.

Many infectious diseases in humans are caused by virulent biofilms, including those occurring in the mouth. Among them, dental caries continues to be one of the most ubiquitous and costly biofilm-dependent oral diseases worldwide, which compromise the health and well-being of children and adults alike (Marcenes et al., 2013). This disease results from complex interactions between specific oral microorganisms, host factors and diet that promote the establishment of caries-producing (cariogenic) biofilms on tooth surfaces (Selwitz et al., 2007; Russell, 2008). The assembly of cariogenic biofilms is a prime example of how pathogenic bacteria such as *Streptococcus mutans* orchestrate the development of virulent biofilms on (tooth) surfaces, as an extracellular matrix assembles (as reviewed in Hamada and Slade, 1980; Loesche, 1986; Bowen and Koo, 2011). EPS are the main constituents of the matrix in cariogenic biofilms and are recognized as essential virulence factors associated with dental caries (Yamashita et al., 1993; Mattos-Graner et al., 2000; Vacca Smith et al., 2007). Nevertheless, other constituents such as extracellular DNA (eDNA) and lipoteichoic acids (LTA) have been also found in high amounts in the matrix of cariogenic biofilms.

The microbial composition and structural organization of cariogenic biofilms are not static but rather change dynamically (Marsh, 2003). In the complex oral microbiome, *S. mutans* is not always the most numerous species; many organisms are equally acidogenic and aciduric (Takahashi and Nyvad, 2011; Valm et al., 2011; Mattos-Graner et al., 2014). However, *S. mutans* is a major matrix producer and can rapidly modulate the formation of cariogenic biofilms when dietary sucrose and starch are present (Firestone et al., 1982; Marsh, 2003; Ribeiro et al., 2005; Paes Leme et al., 2006). Sucrose serves as substrate while starch hydrolysates act as acceptors for EPS (glucans and fructans) synthesis by *S. mutans* glucosyl- and fructosyltransferases (Gtfs and Ftfs) (Fu and Robyt, 1991; Bowen and Koo, 2011). Moreover, *S. mutans*-released Gtfs are constituents of the pellicle and synthesize glucans *in situ*, promoting local colonization of *S. mutans* and other organisms; Gtfs also bind to surface of other oral microorganisms converting them into glucan producers (as reviewed in Bowen and Koo, 2011). Thus, the production of EPS on surfaces enhances local accumulation and clustering of microbes on teeth. As the biofilm develops, the EPS formed *in situ* enmeshes and surrounds the microorganisms while forming an insoluble matrix facilitating the assembly of spatially heterogeneous yet cohesive 3D multicellular structures (as reviewed in Koo et al., 2013).

The spatial heterogeneities shaped by EPS synthesis form a complex 3D matrix architecture and create environmental and protective niches within biofilms that can directly modulate caries pathogenesis. Available evidence suggests there is a substantial limitation of diffusion into and out of the biofilm due to the presence of insoluble EPS-rich matrix, which could facilitate acid accumulation and hinder neutralization by buffering saliva that surrounds the teeth, as reviewed recently (Bowen and Koo, 2011; Koo et al., 2013) and thereby it will not be discussed here. Furthermore, EPS from *S. mutans* may be charged due to the incorporation of LTA (Kuramitsu et al., 1980; Rölla et al., 1980; Vickerman and Jones, 1992) and possibly eDNA (see later). The presence of negatively charged EPS appears to affect the penetration (and antimicrobial activity) of positively charged chlorhexidine into biofilms (Hope and Wilson, 2004). The detailed mechanisms involved in limiting diffusion remain to be elucidated. Furthermore, little is known about how secreted metabolites and proteins migrate from producing microorganisms within the matrix of intact biofilms. It is noteworthy that polysaccharide within plaque-biofilms is not evenly distributed, and its density is enhanced at the tooth interface (Reese and Guggenheim, 2007), which could affect mass transport and diffusion properties across the biofilm structure (Thurnheer et al., 2003; Robinson et al., 2006). Recently, Xiao et al. (2012) showed the importance of the manner by which the EPS matrix is assembled three-dimensionally and how it is spatially arranged with the bacterial cells to create compartmentalized pH microenvironments, while conferring protection to bacteria against chlorhexidine locally within intact biofilm architecture.

In parallel, sugars are fermented by *S. mutans* and other acidogenic organisms embedded in the matrix, facilitating the formation of highly acidic microenvironments (pH 4.5–5.5) (Vroom et al., 1999; Xiao et al., 2012; Guo et al., 2013). The low pH niches induce EPS synthesis while cariogenic organisms such as *S. mutans* prosper (Quivey et al., 2000; Lemos and Burne, 2008; Smith and Spatafora, 2012). As the environmental acidic stress further increases, the microbial diversity is reduced in favor of a highly acid-tolerant and acidogenic microbiota (Takahashi and Nyvad, 2011). Consequently, local acidity ensures continuous biofilm accretion and acid-dissolution of adjacent tooth enamel, leading to the onset of dental caries. Altogether, the creation of localized microenvironments, delineated by a diffusion-limiting matrix, has profound effects on the architecture, metabolism and expression of virulence of biofilm as a whole. Although the immediate cause of enamel dissolution is certainly acid production, the absence of the “sheltering” effect of the biofilm matrix would minimize the ability of acids to demineralize in the presence of saliva. The insoluble EPS-rich matrix produced by *S. mutans* is a unique virulence feature of this species that helps to set it apart from other acidogenic and aciduric species.

Importantly, well-established biofilms become recalcitrant to antimicrobials and difficult to remove from surfaces (Hall-Stoodley et al., 2004; Marsh et al., 2011; Stewart, 2014). Historically (and currently), mechanical removal of plaque-biofilm by tooth brushing and dental flossing, in addition to fluoride use, have been the standard measures to prevent dental caries. Thus, enhanced understanding of how biofilms can be disrupted and removed from the surface of attachment could lead to improved strategies to eradicate them. The EPS formed on surfaces and further development of polymeric matrix may be responsible for the mechanical properties of cariogenic biofilms, such as adhesive strength and cohesiveness.

The presence of glucans enhances local adhesion strength of *S. mutans* on apatitic surfaces (Schilling and Bowen, 1992; Tsumori and Kuramitsu, 1997; Cross et al., 2007), while development of a glucan-rich matrix and cell-glucan adhesions are essential for the structural integrity of the biofilm 3D architecture (Banas and Vickerman, 2003; Lynch et al., 2007; Xiao et al., 2012). In addition, the viscoelastic properties of *S. mutans* biofilms are similar to those of organic polymers (Vinogradov et al., 2004). Thus, the mechanical properties of biofilms may be determined by the EPS composition and degree of branching, and spatial distribution of EPS-rich matrix, which in turn modulate the three-dimensionality of the biofilm architecture. A variety of biophysical methods, ranging from rheometry (Klapper et al., 2002; Towler et al., 2003), uniaxial compression to fluid flow (Körstgens et al., 2001; Busscher and van der Mei, 2006), and atomic force spectroscopy (Cross et al., 2007; Das et al., 2011) have been applied to characterize the mechanical properties of bacterial adhesion and biofilm formation. However, it remains unclear how EPS modulate adhesive and cohesive forces of the matrix, which are essential for the mechanical stability and surface attachment of biofilms, particularly in cariogenic biofilms where the EPS matrix plays a critical scaffolding role.

Recently, we investigated the mechanical stability and surface detachment of mature *S. mutans* biofilms using rheometry and shear stress-based methods. A rheometer measures the changes in rigidity and viscoelasticity of biofilms, which are key parameters of their mechanical properties (Vinogradov et al., 2004; Cense et al., 2006; Cheong et al., 2009). However, biofilm's response to fluid shear stress (deformation and detachment) is also recognized as relevant for its mechanical stability (Klapper et al., 2002). We developed a device that generates a diverse range of shear stress to assess how incremental increases in shear cause distinctive pattern of biofilm removal and detachment from saliva-coated hydroxyapatite (sHA) surfaces (which mimics pellicle-coated teeth) (Hwang et al., 2014). We used the shear-inducing device with EPS-digesting enzymes to examine the influence of the matrix on mechanical stability of well-established *S. mutans* biofilms (Hwang et al., 2014).

The data revealed a two-phase biofilm removal profile from the sHA surface following measured applications of increasing shear stresses. We observed an initial bulk elimination that was proportional to the amount of shear stress applied, followed by increased resistance to removal of the remaining biomass close to the surface. Confocal fluorescence imaging showed a thick and dense basal layer of EPS adjacent to the sHA surface, which could enhance biofilm anchoring to the surface and thereby contribute to the increased resistance to shearing. Indeed, degradation of the EPS via non-biocidal glucanohydrolase (dextranase) caused a major disruption on the ability of the biofilm to withstand mechanical removal from the surface, greatly facilitating its removal and further surface detachment (Hwang et al., 2014). Strikingly, large sections of the biofilms were completely detached from the sHA surface upon application of shear stress on dextranase-treated biofilms. Thus, both the content and spatial distribution of EPS-rich matrix influence the mechanical stability and surface attachment of intact *S. mutans* biofilms.

To further investigate the role of EPS-matrix on the mechanical stability of the biofilms, the rheological properties of biofilms treated with EPS-digesting enzymes were examined using rheometry on intact biofilm samples (Mert and Campanella, 2008; Figure 1). Intact *S. mutans* biofilms presented higher storage modulus (31,718 ± 3,440 Pa) than loss modulus (3,775 ± 450 Pa), indicating that *S. mutans* biofilm has a viscoelastic (solid-like) behavior and is highly structured (Hwang et al., 2014), in agreement with previous studies (Vinogradov et al., 2004; Cense et al., 2006). The contribution of EPS matrix to the viscoelasticity and rigidity of *S. mutans* biofilm was assessed by treating biofilms with two distinct glucanohydrolases: mutanase, which hydrolyzes α-(1→3) glucosidic linkages and branch points in GtfB and GtfC-derived insoluble glucans, and dextranase, which digests α-(1→6) (and branch points) present in GtfB/C- as well as in GtfD-derived glucans (Guggenheim, 1970; Hayacibara et al., 2004). The insoluble glucans produced by GtfB and C are essential for the assembly of a 3D extracellular matrix scaffold and localized pH microenvironments in cariogenic biofilms (Xiao et al., 2012), which may explain their role in the expression of *S. mutans* virulence *in vivo* (Tanzer et al., 1985; Yamashita et al., 1993) as well their association with caries activity in humans (Mattos-Graner et al., 2000; Vacca Smith et al., 2007).

**Figure 1:**
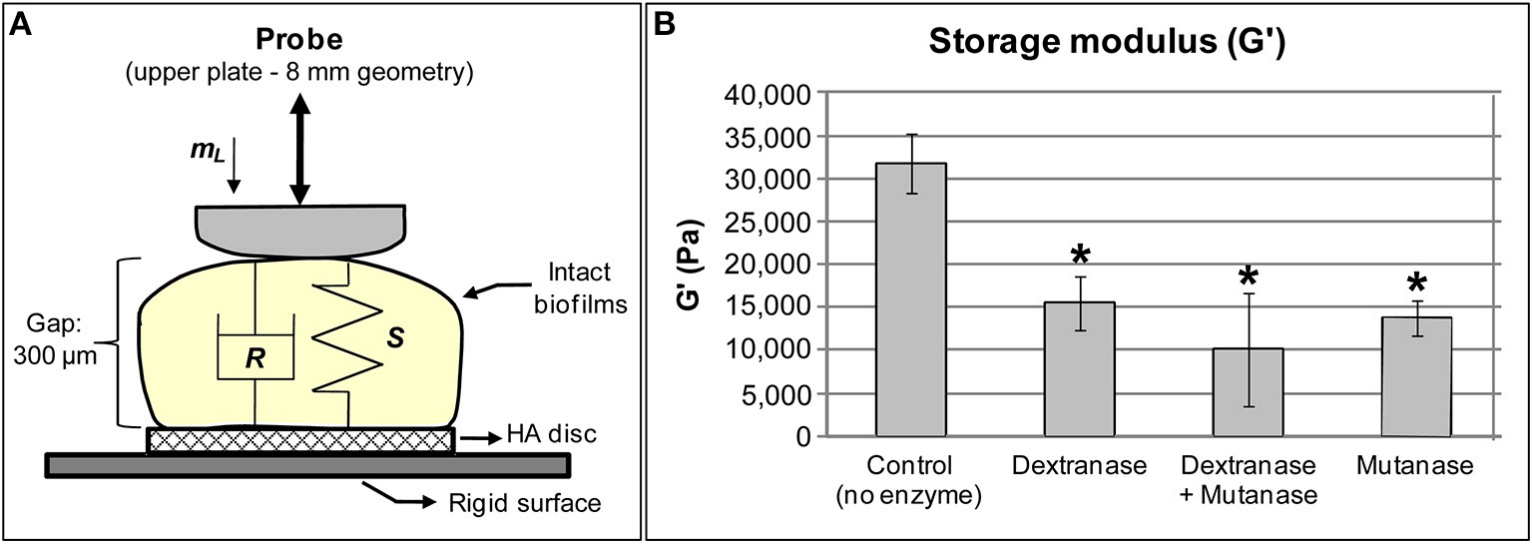
***Streptococcus mutans* biofilms subjected to rheometry analysis. (A)** Close-up view of biofilm samples placed between the upper and bottom plates of the rheometer. Stiffness, S, indicates rigidity of the sample whereas damping, R, is related to viscosity of the samples. *m_L_* is the initial load applied to the sample during measurement. Gap: the size was standardized at 300 μm because the 3D structure of biofilm is not homogeneous, and 300 μm was the average maximum thickness of 115 h-old biofilms grown in 1% sucrose. **(B)** *Storage modulus* of *S. mutans* biofilms incubated with 2 units of each enzyme (^*^P < 0.05 vs. control), using small amplitude oscillatory shear experiments performed within the linear viscoelastic region at 1 Hz. All treatments with glucanohydrolases modified the rheological properties, specifically decreased the *storage modulus* of treated biofilms.

Digestion of EPS matrix with dextranase or mutanase caused more than a two-fold reduction in the storage modulus (Figure 1) compared to untreated biofilms, while the combination of both glucanohydrolase led to a 3-fold reduction (vs. untreated biofilms). Treatment with glucanohydrolases substantially changes biofilm rigidity, which greatly facilitated removal of mature biofilms (Hwang et al., 2014). These observations indicate that the digestion of α-(1→6) and α-(1→3) glycosyl linkages in the matrix structure severely impacts biofilm cohesiveness and stability. Indeed, the 3D biofilm structure eventually collapsed when the biofilms were incubated with the EPS-digesting enzymes for a prolonged period (>5 h). Altogether, the ability of mature *S. mutans* biofilms to withstand mechanical clearance is associated at least in part by the amounts, spatial distribution and structural rigidity of the exopolysaccharides-rich matrix.

These findings have clinical relevance in the pathogenesis of dental caries and development of novel antibiofilm approaches. The shear rate in the oral cavity due to salivary flow is relatively low (Bourne, 2002), which could explain why EPS-mediated biofilm build-up persists on tooth surfaces and is difficult to detach under salivary flow. Thus, novel devices to enhance mechanical removal of biofilms can be developed based on knowledge about the biophysical properties of the biofilm. Recently, a prototype AirFloss instrument that generates high shear stress locally was capable of removing sucrose-grown *S. mutans* biofilms from the interproximal space (Rmaile et al., 2014). Furthermore, enzymes or compounds capable of altering the viscoelastic properties of the biofilm could be effective to prevent biofilm-dependent diseases (Daniels et al., 2010; Kostakioti et al., 2013; Nguyen et al., 2014). The possibility of using glucanohydrolases as therapeutic approach against dental caries has been explored (e.g., Bowen, 1972; Guggenheim et al., 1980) despite limitations in the clinical setting, possibly due to lack of retention in the mouth and/or enzyme degradation by proteolysis in saliva (Hull, 1980). Nevertheless, the concept of digesting/removing or changing the structure of EPS matrix to control biofilms is certainly attractive. Thus, new enzymes or novel approaches to deliver or retain them in active form should be devised.

Clearly, EPS play a critical role in the assembly and virulence of cariogenic biofilms by providing a 3D scaffold that shape the microenvironment and ensure mechanical stability. Yet, they are not alone. The production and release of both eDNA and LTA by *S. mutans* is also highly induced by sucrose, starch and increased acidity (Ciardi et al., 1977, 1981; Jacques et al., 1979; Rölla et al., 1980; Hardy et al., 1981; Perry et al., 2009; Klein et al., 2010). However, their functional roles in matrix assembly remain poorly understood.

## eDNA and LTA also influence the structure and stability of cariogenic biofilm matrix

eDNA and LTA may contribute to the assembly of the matrix by enhancing glucan synthesis (Kuramitsu et al., 1980; Chiu and Baker, 1994) and promoting bacterial binding to surfaces (Ciardi et al., 1977; Vickerman and Jones, 1992; Das et al., 2010). eDNA is often a byproduct of autolysis (Steinberger and Holden, 2005; Allesen-Holm et al., 2006; Perry et al., 2009), but it can be also secreted via microvesicles or membrane vesicles (Liao et al., 2014). eDNA enhances *S. mutans* adhesion to glass surfaces by creating thermodynamically favorable conditions for bacterial adhesion and surface aggregation due to acid-base interactions (Das et al., 2010). Moreover, eDNA builds and strengthens the matrix by interacting with exopolysaccharides within *Myxococcus xanthus* biofilms, where acidic pH enhances the binding between eDNA and EPS (Hu et al., 2012). These findings may be relevant to cariogenic biofilms.

Dietary sucrose and starch enhance release of eDNA into the matrix in high quantities due to upregulation of *lytTS* genes (and the *ccpA* gene) during *S. mutans* biofilm formation (Klein et al., 2010). The two component system *lytTS* is required to activate expression of *lrgAB* genes that are part of *S. mutans* arsenal to control autolysis and biofilm formation (Ahn et al., 2010). The expression of *lytTS* and *lrgAB* is regulated by availability of carbohydrates via CcpA (Ahn et al., 2010). In addition, the *gtfB* expression is also upregulated in biofilm growing in the presence of sucrose and starch (Klein et al., 2009, 2010). Thus, eDNA interacting with GtfB, may have important roles in EPS production, *S. mutans* colonization and biofilm matrix assembly (Klein et al., 2010; Liao et al., 2014).

**Figure 2:**
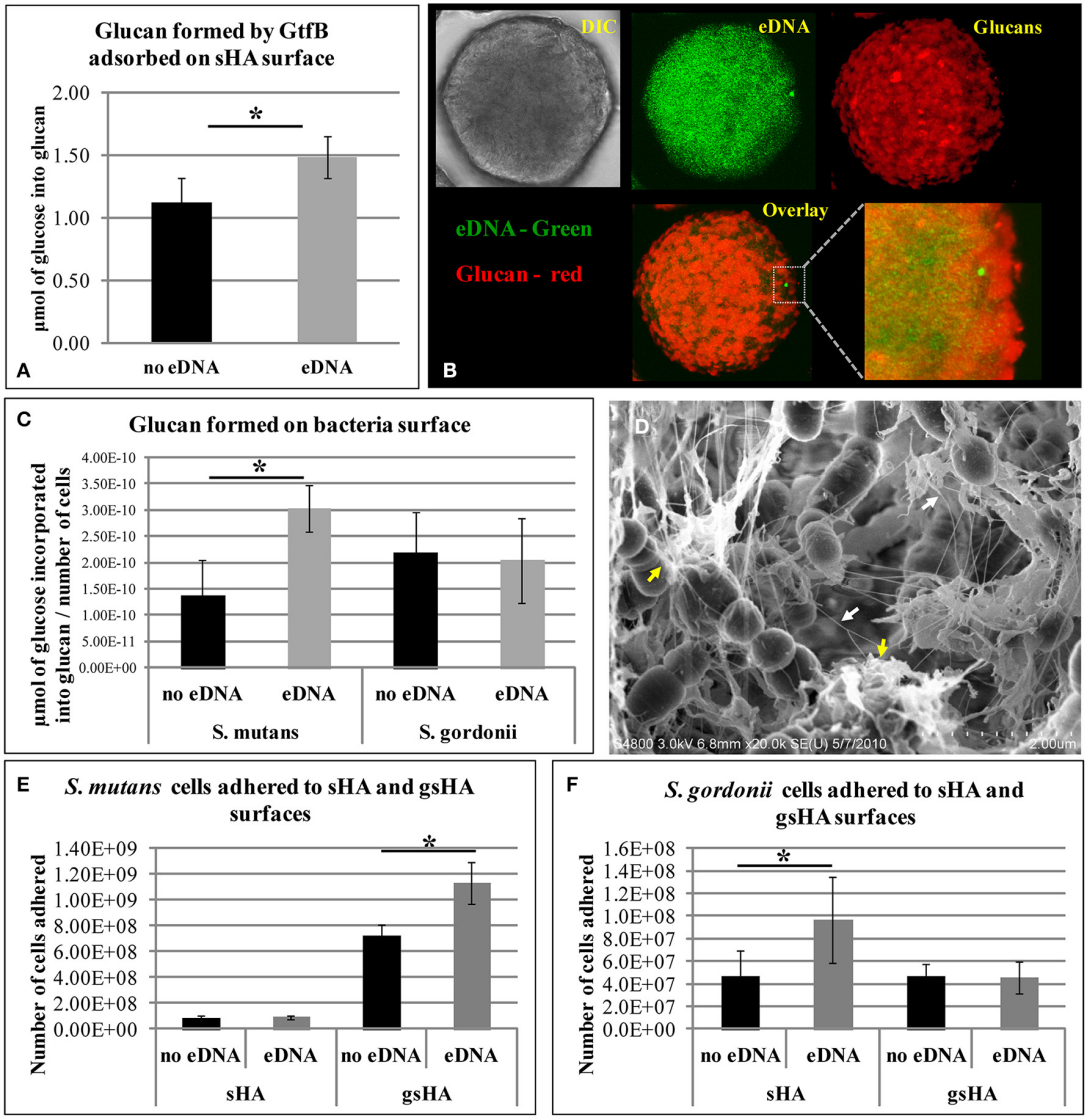
**Influence of eDNA on glucans synthesis and bacterial adhesion. (A)** Glucan formed by GtfB adsorbed to sHA surface. **(B)** This panel of images shows the sHA bead in DIC (gray), in green is the eDNA associated to surface (which presents a punctuated distribution pattern), and red are the glucans formed. Overlay and close-up image show eDNA interspersed with glucans. **(C)** Glucan produced by GtfB adsorbed to *S. mutans* and *S. gordonii* cells. **(D)** FE-SEM analysis of *S. mutans* biofilms on apatitic surface. Images highlight interaction of nanofibrous eDNA (white arrows) and wool-like glucans (yellow arrows). Bacterial adhesion to apatitic surfaces in presence and absence of eDNA are shown for *S. mutans* **(E)** and *S. gordonii* **(F)**. sHA: saliva-coated hydroxyapatite; gsHA: glucan formed on saliva-coated hydroxyapatite. An asterisk (^*^) denotes *p* < 0.05. Note: **(D,E)** were kindly provided by Dr. Zezhang (Tom) Wen (School of Dentistry, Louisiana State University Health Sciences Center, New Orleans, Louisiana, USA).

eDNA increased glucan synthesis by GtfB adsorbed on saliva-coated hydroxyapatite (sHA) and on *S. mutans* cell surfaces (but not on *Streptococcus gordonii*), while being incorporated into glucan structure (Figure 2). Moreover, a larger number of *S. mutans* cells bound to the glucan formed on apatitic surface in presence of eDNA than did on glucan without eDNA, while no effects were observed on bacterial binding to sHA (Liao et al., 2014; Figure 2). eDNA interspersed with glucans may provide enhanced binding sites for *S. mutans* colonization. Strikingly, an opposing trend was observed for *S. gordonii* (a commensal non-cariogenic organism), which adhered more avidly on sHA with eDNA than on glucan-coated surface either with or without eDNA (Figure 2). Therefore, eDNA may contribute to cariogenic biofilm initiation by increasing EPS (glucans) synthesis *in situ* that display selective and enhanced binding capacity to *S. mutans*.

Furthermore, as biofilms develop, eDNA appears to be incorporated into the matrix because the addition of DNAse disrupted the structural integrity of the biofilms (Klein et al., 2010; Liao et al., 2014), suggesting that eDNA play an important role in biofilm matrix structure. Further biofilm characterization confirmed that eDNA is indeed an essential component of the matrix, which is found interconnected with glucans (Figure 2). It is apparent that eDNA is released not only from cell lysis, but also through membrane vesicles during different phases of biofilm development (Liao et al., 2014). Deficiency of protein secretion and membrane protein insertion machinery components (i.e., Ffh, YidC1, and YidC2) can reduce the amount of eDNA in the matrix (Liao et al., 2014). Thus, eDNA together with GtfB-derived glucans may facilitate accumulation of cariogenic bacteria while assembling highly structured and cohesive biofilm matrix.

Lipoteichoic acids may be also relevant for matrix assembly and biofilm formation. LTA from *S. mutans* is abundant in the pellicle and induces insoluble glucan synthesis (Ciardi et al., 1977; Kuramitsu et al., 1980; Rölla et al., 1980). LTA may enhance bacterial binding to tooth surfaces, and affect the composition of the matrix (Kuramitsu et al., 1980; Rölla et al., 1980), particularly when sucrose and starch are available. LTA is anchored to the cell membrane (Ellwood and Tempest, 1972; Neuhaus and Baddiley, 2003) and can be released to the extracellular milieu during cell wall remodeling. The mechanism of LTA synthesis was described for a few species but not for *S. mutans* (Reichmann and Gründling, 2011; Denapaite et al., 2012). Our BLAST analyses showed that *S. mutans* gene SMU.775 (a hypothetical protein) is a homolog to *ltaS* gene that encodes the LTA synthase enzyme of *Staphylococcus aureus*. The *dltABCD* operon is required for addition of D-alanine residues to LTA (Neuhaus and Baddiley, 2003); these residues affect adhesion, biofilm formation and cariogenicity of *S. mutans* (Spatafora et al., 1995, 1999; Gross et al., 2001; Götz, 2002). Furthermore, the proteins encoded by genes *dltA, dltD* and SMU.775 were most abundant when the matrix is being actively constructed during the initial phases of biofilm formation (Klein et al., 2012).

It is apparent that eDNA and LTA may act in concert with EPS, directly modulating the assembly, structural organization and functional properties of the matrix during cariogenic biofilm formation; alterations in these processes can alter the matrix composition impacting biofilm assembly and virulence potential. Furthermore, the production of *S. mutans* eDNA and LTA is enhanced in the presence of other organisms (Klein et al., 2012). Thus, other species may contribute to the matrix construction, either by directly modulating *S. mutans* expression of gene products involved with eDNA and LTA export, or by releasing LTA and eDNA themselves into the matrix. The mechanisms that triggers the release of eDNA and LTA and how these byproducts are incorporated into the biofilm matrix remain unclear, and studies are in progress to investigate their structural and functional roles in cariogenic biofilms.

## Conclusions and future perspective

The matrix in cariogenic biofilms has roles far beyond providing bacterial binding sites and holding microbial cells together. It provides a diffusion-limiting 3D scaffold that shapes the spatial and microenvironmental heterogeneities, helping to create a myriad of acidic pH and protective niches, while modulating the mechanical properties of the biofilm. These properties are critical for the persistence, dynamic microbial composition changes and expression of virulence of cariogenic biofilms. In this context, it is conceivable that the primary role of *S. mutans* in the pathogenesis of dental caries resides with its ability to assemble an insoluble polymeric matrix and not simply with numerical superiority or acidogenicity. Furthermore, the presence of other organisms in the biofilms may also contribute to the matrix assembly. Clearly, enhanced understanding about the extracellular matrix biology and its functional properties may lead to enhanced ways to prevent dental caries. For example, how other components of the matrix (such as eDNA and LTA) are associated with glucans, and how together affect the microenvironmental and mechanical properties of cariogenic biofilm need further clarification. At the same time, measurement of biophysical properties associated with resistance to mechanical clearance may be additional factors to be considered when searching for effective antibiofilm therapeutics. The results of such investigations may have relevance beyond the mouth, as matrix and microenvironmental niches hinder drug efficacy in other biofilm-associated diseases and industry related issues.

### Conflict of interest statement

The authors declare that the research was conducted in the absence of any commercial or financial relationships that could be construed as a potential conflict of interest.
